# Centering Lived Experience in Developing Digital Interventions for Suicide and Self-injurious Behaviors: User-Centered Design Approach

**DOI:** 10.2196/31367

**Published:** 2021-12-24

**Authors:** Kaylee Payne Kruzan, Jonah Meyerhoff, Candice Biernesser, Tina Goldstein, Madhu Reddy, David C Mohr

**Affiliations:** 1 Center for Behavioral Intervention Technologies Feinberg School of Medicine Northwestern University Chicago, IL United States; 2 Department of Psychiatry University of Pittsburgh Medical Center Pittsburgh, PA United States; 3 Department of Informatics Donald Bren School of Information and Computer Sciences University of California, Irvine Irvine, CA United States

**Keywords:** user-centered design, intervention, suicide, nonsuicidal self-injury, lived experience, technology-enabled services, digital intervention, engagement, mobile phone

## Abstract

**Background:**

The prevalence of self-injurious thoughts and behaviors (SITB) signals a growing public health crisis. Despite a recognized need for improved and scalable interventions, the field of SITB intervention faces several challenges: existing interventions are often time and resource intensive, most individuals with SITB do not seek formal mental health care, and efficacious treatments are characterized by small effects. Combined, these challenges indicate a need for improved SITB interventions for individuals in formal treatment and those who are not treatment engaged but are at high risk of worsening mental health and future suicide attempts.

**Objective:**

We present a methodological approach and set of techniques that may address these challenges by centering the lived experience of individuals with SITB in the process of developing needed services: user-centered design (UCD).

**Methods:**

We highlight the value of UCD in the context of digital interventions for SITB by describing the UCD approach and explicating how it can be leveraged to include lived experience throughout the development and evaluation process. We provide a detailed case example highlighting 3 phases of the early development process that can be used to design an intervention that is engaging and meets end-user needs. In addition, we point to novel applications of UCD to complement new directions in SITB research.

**Results:**

In this paper, we offer a 2-pronged approach to meet these challenges. First, in terms of addressing access to effective interventions, digital interventions hold promise to extend the reach of evidence-based treatments outside of brick-and-mortar health care settings. Second, to address challenges related to treatment targets and engagement, we propose involving individuals with lived experience in the design and research process.

**Conclusions:**

UCD offers a well-developed and systematic process to center the unique needs, preferences, and perceived barriers of individuals with lived SITB experience in the development and evaluation of digital interventions.

## Introduction

### Background

Suicide is a leading cause of death globally [1], and international rates of nonsuicidal self-injury (NSSI) are also high, with community (nonclinical) prevalence of 17.2% for adolescents, 13.4% for young adults, and 5.5% for those beyond young adulthood [2]. Lifetime prevalence of suicidal ideation is estimated to be between 22.3% and 32.7%, with 12-month prevalence of 10.6% in young adults [3,4]. Upward trends in these rates signal a growing public health crisis.

Despite significant advances in our understanding of the epidemiology and phenomenology of self-injurious thoughts and behaviors (SITB), including suicide and NSSI, several key challenges exist for the field of SITB intervention. First, existing SITB interventions are often time and resource intensive, making them inaccessible to many, and difficult to scale to meet the large and growing need. Second, most individuals with SITB are hesitant to disclose their struggles and do not seek formal mental health care [5-7]. These challenges reflect a need to improve interventions for individuals who make contact with formal treatment settings as well as for those individuals who are not, and do not wish to be, treatment engaged.

Digital mental health interventions (DMHIs) are a promising and viable option to address these challenges because they are efficacious, scalable, and flexible enough to be used within, as an adjunct to, or independent of, formal mental health services. DMHIs typically refer to technology-based interventions that are patient facing. However, how DMHIs are deployed can vary significantly [8]. For example, DMHIs include stand-alone tools that are self-contained and used by patients for self-management as well as adjunctive tools meant to augment patients’ engagement in face-to-face treatments. DMHIs also vary along a spectrum of human support from fully automated or self-guided tools to those that incorporate digital coaching or lightweight human support to ensure effective use, reduce barriers, fortify points of disconnection [9], and ensure stronger adherence to the tool.

A recent systematic review of DMHIs for SITB found 22 eligible trials: 12 randomized controlled trials (RCTs), 9 single-arm trials, and 1 crossover counterbalanced controlled design [10]. The outcomes included suicidal ideation (n=14), suicide attempt (n=3), NSSI (n=4), and self-injurious behaviors not otherwise specified (n=2). Because of small sample sizes for most outcomes, a meta-analysis was only conducted for suicidal ideation, and the effect size was small (g*=*–0.12, 95% CI –0.29 to 0.05) for DMHI conditions compared with control conditions. This is similar to trials of standard face-to-face treatments, which have also produced small effects for suicidal ideation (g*=*–0.09, 95% CI –0.15 to –0.02) and SITB outcomes combined (g*=*–0.17, 95% CI –0.22 to –0.12) [11]. As most DMHIs are based on these evidence-based treatment models with small effects, this likely limits the potential effectiveness of DMHIs for SITB. Although incorporating user-centered design (UCD) into DMHIs for SITB alone is unlikely to sufficiently address the issue of universally small effect sizes among interventions for SITB, UCD provides a set of methodologies to refine and identify new targets and ensure that interventions are designed to meet end-user needs. Improved alignment between intervention targets and clinical needs and delivery of clinical interventions in forms more acceptable to users can lead to improved engagement—an important potential driver of clinical change [12].

Although studies of DMHIs for SITB have described good patient-reported acceptability and initial engagement with these tools, a rapid decline in engagement is common. This may suggest that although people were interested in receiving help through DMHIs, their experience with these products did not meet their expectations or preferences. Engagement with DMHIs has been a challenge across the field of digital mental health [13,14].

### Stakeholder Input

To address the challenges of declining DMHI engagement and the stalled effectiveness of existing NSSI and suicide prevention interventions, we suggest including stakeholder input, particularly from individuals with lived experience, in the SITB DMHI design and evaluation process. This will ensure that we are designing highly engaging and effective interventions that focus on broadly applicable treatment targets. Given the sensitivity of working with a population at heightened risk of suicide, there is a need for rigorous ethical review of all study and risk management protocols [15]. Although there has been growing interest in research that incorporates the voices and needs of individuals with lived SITB experience [16,17], there has been little guidance on how best to do so.

In this paper, we describe UCD methodologies as a means to incorporate lived experience in the research process to develop interventions that are highly attuned to the needs of the individuals they are meant to support. Our aims are to (1) describe the UCD approach, (2) show how it can be leveraged to include lived experience throughout the development and evaluation process through a case example, and (3) point to promising opportunities to integrate lived experiences in research processes for SITB interventions.

## Methods

### Procedures

In this paper, we illustrate the process of using UCD with individuals with lived experience of SITB. This work was guided by a review of selected works on the design and development of DMHIs for SITB in the existing literature. For simplicity, we chose to illustrate the UCD process in the context of the development of a single DMHI targeting SITB. To select an appropriate case example, multiple coauthors reviewed the literature on DMHI for SITB and considered innovative methods, intervention elements, UCD technique examples, and attention to safety and ethics. The Brite app, a suicide prevention smartphone app that was developed and tested through an RCT at the University of Pittsburgh and University of Texas Southwestern Medical Center, was ultimately chosen as our case example given the aforementioned criteria [18,19]. In the next sections, we discuss the UCD process and prior use of UCD for SITB interventions; subsequently, we present a detailed case example through the development and evaluation of Brite.

### What Is UCD?

UCD is an approach that grounds the development of new DMHIs in the specific needs, challenges, and preferences of stakeholders and end users. In the context of DMHIs for SITB, stakeholders may include policy makers, clinicians, caretakers, and friends of individuals with SITB. End users are defined as the individuals whom the intervention is intended to serve and support, for example, individuals with SITB, for patient-facing DMHIs and clinicians or coaches for adjunctive tools. This inclusion of various stakeholders and end users in the design process is characteristic of UCD and has been used to improve the efficacy and acceptability of services.

The key aims of UCD are to increase the usability (ease of use) and usefulness (the extent to which it assists users in achieving their goals) of a technology [20]. UCD also seeks to improve satisfaction with a technology by making it acceptable and more engaging to end users. Although UCD methods are often applied to technology-based products for use in traditional work environments, they have recently been applied to many different contexts and for novel nontechnology purposes, including improving psychotherapy [21], medical care [22], and implementation strategies [23,24]. In the context of DMHIs, UCD has been leveraged to develop tools that closely align with users’ needs, ensure inclusion of the types of content and functionality that users expect, and ensure that the intervention is delivered in an appealing and usable format to seamlessly fit into their lives [25-27].

### A UCD Process

**Figure 1:**
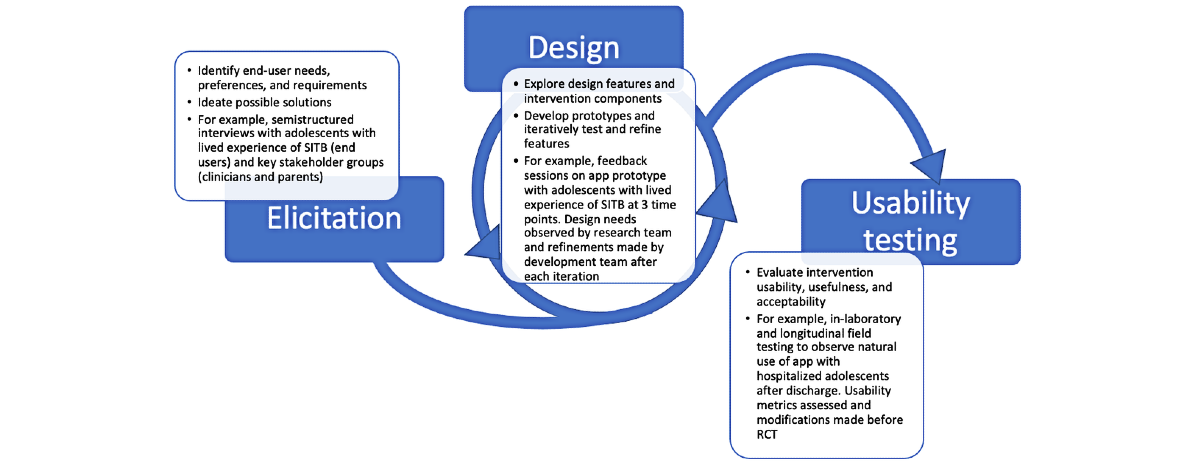
User-centered design process for a self-injurious thoughts and behaviors intervention for adolescents after hospital discharge. RCT: randomized controlled trial; SITB: self-injurious thoughts and behaviors.

As a methodological approach, UCD typically includes several iterative phases (Figure 1) that are either formative or summative [20]. Formative UCD processes, including elicitation and design activities, produce an initial version of the intervention, whereas summative processes evaluate the usability of the intervention. Within each phase, different objectives are met using UCD techniques (Table 1). The *elicitation phase* is focused on understanding the specific needs, preferences, limitations, and requirements of end users by directly engaging them in elicitation activities such as interviews, focus groups, or observational techniques. This phase aims to identify, and brainstorm possible solutions to, needs or challenges in direct collaboration with the population of interest. At the end of the elicitation phase, researchers compile the needs and requirements of end users in a design document, which can be used to develop prototypes for presentation to users again in design-focused activities.

The *design phase* begins the development of the DMHI and involves a set of iterative design feedback activities with end users, often involving *prototypes*—tools that enable feature and service ideation and initial formative evaluation of the proposed technology’s functions and features. Prototypes approximate a feature or several features of an intervention and can range from paper depictions of an app interface to a wireframe or low-fidelity (ie, alpha) version of the app itself. After each design feedback session, improvements are made to the prototype so that it gets progressively closer to meeting end users’ needs in its most acceptable form.

Finally, *usability testing* focuses on verifying that the final (ie, beta) version of the DMHI meets the requirements of the end users through single-session or longitudinal usability testing. Ideally, this phase includes both qualitative and quantitative data collection, as well as testing the intervention in the field. Data from this phase will inform intervention refinements before it is ready for initial pilot, feasibility, or clinical outcomes testing.

Table 1 lists examples of common UCD techniques used in formative and summative evaluation. Any single technique can be used for different purposes. For example, although we list focus groups under elicitation-focused techniques, for its use in identifying user needs and preferences, it can also be used in the design phase to ideate or identify usability issues. Techniques can also be combined.

**Table 1:** User-centered design techniques.

Technique				Brief description		Example for SITB^a^ research
**Formative evaluation**						
	**Elicitation-focused techniques**					
		Card sorting	Sort a set of cards with constructs relevant to the intervention into groups that make sense to the end user		Individuals with lived SITB experience sort emotion regulation features in terms of the most to least important for SITB management	
		Concept mapping	Create an illustration mapping the relationship between the research question and related concepts		Individuals with lived SITB experience create a pen-and-paper map of emotions, thoughts, and contexts and how these factors relate to certain coping strategies	
		Diary or EMA^b^ studies	Prompt end users to share relevant details about their perception or experience at scheduled or random intervals over time		Individuals with lived SITB experience are prompted 3 times daily to understand contexts or environments associated with heightened suicidal or self-injurious thoughts	
		Focus groups	Stakeholders meet for a moderated discussion related to the research question		Clinicians, parents, and young people with SITB are gathered to discuss day-to-day needs	
		Interviews	End users meet with the researcher for a structured or semistructured interview		Interviewing individuals with current SITB to understand how they conceptualize and engage in SITB self-management and identify needs [28]	
		Personal inventories	End users describe and share artifacts that are personally valuable or relevant in their life		Individuals with lived SITB experience are asked to bring to an interview session the personal items that provide them hope or comfort when times are difficult	
		Task analysis	Identify steps that the end user takes to complete a task		Individuals with lived SITB experience are asked to access and practice coping tools in an app	
	**Design-focused techniques**					
		Co-design workshops	Designers, end users, and stakeholders meet to collaborate on the design of an intervention		Individuals with lived experience of SITB and clinicians meet with the purpose of ideating and designing prototypes that will help end users when they experience urges to self-injure [29]	
		Passive storyboards	End users are presented with scenarios—or narratives describing a set of contexts and actions—and probed for how they would engage or navigate the scenario		In the presence of qualified practitioners, individuals with lived SITB experience are asked to reflect on a situation when they experienced an urge to self-injure and asked how they managed the urge and what resources they would usually use	
		Interactive storyboards	End users are presented with a narrative of the intervention in a series of panels and participate in the narrative while being probed on whether the product or solution meets requirements		Individuals with lived SITB experience are provided a visual narrative of a proposed intervention that aims to intervene in moments when suicidal distress occurs and are asked to consider its acceptability through each interaction	
		Blue sky prototyping	Considering designs that were previously ignored because of feasibility (eg, cost, risk, or complexity) to encourage radical innovation and creativity		Through a focus group, clinicians are provided with an implementation plan for a suicide prevention intervention that they believed had liability concerns. Rather than disregarding it, they brainstormed implementation strategies to mitigate their concerns	
		Parallel prototyping	Multiple design concepts are embodied and compared concurrently. Parallel prototyping can help provide critical feedback for concept selection		Individuals with lived SITB experience are provided with similar variants of prototypes that differ by look and feel and are asked to describe their perceived acceptability in moments of suicidal crisis	
		Wizard of Oz-ing	A prototype to simulate the full functionality of the intervention with a human operating behind the scenes so that all interactions seem to be computer-driven		Individuals with lived SITB experience are provided with an app prototype and are asked to interact with the app while a researcher navigates display behind the scenes	
**Summative evaluation**						
	**Usability testing techniques**					
		Cognitive walkthrough	End users are presented with an intervention design and asked to show how they would use it based on design features and prompts within the intervention		Individuals with lived SITB experience are presented with screenshots from a web-based program designed to teach distress tolerance and are asked to describe how they would navigate the screen based on prompts	
		Think-aloud exercises	End users engage with the intervention and are asked to speak aloud when they complete a task		Individuals with lived SITB experience interact with an avatar-and-text interface and are asked to speak aloud when they make decisions about how and when to engage [30]	
		Heuristic evaluation	Designers or researchers evaluate prototypes to identify potential usability problems based on heuristics		Clinicians are invited to evaluate early prototypes to determine whether the intervention would be useful and usable for patients with SITB [31,32]	
		Laboratory-based usability testing	End users identify features or interactions that are not usable and are in need of refinement while using the product in a laboratory setting		Individuals with lived SITB experience are brought into the laboratory to engage with the DMHI^c^ to identify issues	
		Field testing	End users engage with a prototype in natural context (eg, daily life and inpatient unit)		Individuals with SITB use the DMHI over the course of the planned length of an intervention (eg, 8 weeks) and respond to usability measures across this period	

^a^SITB: self-injurious thoughts and behaviors.

^b^EMA: ecological momentary assessment.

^c^DMHI: digital mental health intervention.

We note that there are ethical considerations with applying some of these techniques in populations with lived experience of SITB. Although infrequent, some participants may experience distress in relaying information about their lived experience. However, researchers should take measures to mitigate this risk, including following existing guidance on working with populations at elevated risk of suicide [33], guidance on mitigating risk in studies using ecological momentary assessment (EMA) or real-time monitoring [34,35], and guidance on collaborating with institutional review boards to develop protocols that appropriately account for beneficence and well-being [15]. This should first include attention to informed consent and enrollment processes that ensure that participants have the capacity to safely participate in research and that participants are informed of their right to discontinue study participation at any time or skip over questions that they do not wish to answer. During the conduct of research, detailed safety protocols are essential, including procedures for assessing and managing suicidal risk. When conducting UCD work with this population, we recommend that a qualified and adequately trained mental health professional be accessible to participants during all focus groups, interviews, and workshops and be available for consultation in emergent situations. In addition, all participants should be provided with readily accessible emergency contact information throughout the study period in the event that they feel unsafe or have a psychiatric emergency. If a study lasts longer than one session, it is advisable to remind participants of their rights to withdraw from the study and to converse with a qualified professional. In addition, research should be conducted within an environment where people with lived experience feel comfortable and safe sharing their stories. This might involve reviewing rules for confidentiality within focus groups and training research staff to use nonstigmatizing language in the discussion and reporting of SITB (eg, the American Association of Suicidology has a set of guidelines on appropriate language [36]).

### Applications of UCD for a Digital Mental Health and SITB Intervention

Although UCD techniques have been used in the design of DMHIs for SITB, rarely is the full UCD process, including the 3 phases and elements of formative and summative evaluations, used. Practical constraints and a lack of understanding of how to integrate UCD throughout the full design to implementation process are possible factors for the underuse of UCD in clinical intervention design. A main deterrent is the time investment and additional considerations necessary for safely and ethically including stakeholders. Although UCD requires an upfront investment, this formative research should result in a better product that is acceptable to, and engaging for, end users [37]. This formative work can also elicit critical information about the barriers to use and use contexts, which can improve design and implementation.

Some of the more robust examples of UCD for DMHIs for SITB have resulted in well-accepted and usable products. For example, Dimeff et al [30] designed the *Dr Dave* avatar system—which includes patient- and provider-facing components—to reduce hospitalization after emergency department visits. In the patient-facing tool, *Dr Dave* administers the Virtual Collaborative Assessment and Management of Suicidality and asks clarifying questions in a conversational format. For the provider-facing tool, the avatar system summarizes the assessment results for emergency department physicians and is meant to complement standard clinical interviews to optimize patients’ clinical outcomes. Formative elicitation work, focused on understanding workflows and end-user needs, was conducted with hospital administrators, peer specialists, medical providers, and adolescents with suicidal thoughts or behaviors. The findings were then used to develop and iteratively refine prototypes in consultation with stakeholders. Usability and feasibility tests found the final prototype to be acceptable and easy to use.

Czyz et al [38] similarly engaged in iterative development of a text-based intervention to encourage safety plan adherence after hospitalization. Messages were developed through theory and expert opinion, after which adolescents with SITB provided feedback to revise content and language in person and through a longitudinal field study. The intervention was perceived to be acceptable, and a follow-up pilot trial supported its benefit in combination with motivational interviewing–enhanced safety planning [39].

In sum, although UCD techniques have been applied in research on suicide prevention, their application has been limited, and there are no easy-to-follow, comprehensive works that provide guidance on how to incorporate UCD for SITB intervention development. As research teams using UCD often publish different phases of the process across multiple manuscripts, it is often difficult to understand the entire UCD process for any particular digital intervention. In the next section, we provide a comprehensive and detailed example of this process.

## Results

### An Example: Development of a Smartphone App for Adolescents With Suicidal Thoughts or Behaviors

We now elucidate what a robust application of UCD in the development of an SITB intervention could look like through a brief description of the aims of each stage and a detailed case example. The example is based on the development of a suicide prevention intervention featuring an emotion regulation and safety planning smartphone app, Brite, designed for adolescents aged 12-17 years who were hospitalized for suicidal ideation and behavior [18,19]. The intervention aims to reduce suicidal risk during the transition from inpatient to outpatient care, a critical high-risk period for suicidal behavior. The following example includes descriptions of the UCD processes used, augmented by hypothetical elements to demonstrate the wide range of UCD techniques available.

### Phase 1: Elicitation

#### Overview

The aims of the elicitation phase are 2-fold: (1) identify user needs, preferences, and goals and (2) ideate possible solutions. Researchers work with individuals with lived SITB experience to understand their needs and preferences. This might include understanding both how they manage SITB symptoms and salient aspects of their experience, as well as exploring the types of technologies that are acceptable and the services (eg, intervention components) needed or desired as well as those that are unwanted. Questions or interactions for this phase are often informed by existing theory and empiricism on the topic but are not constrained by them. Researcher reflexivity—which entails examining and holding an awareness of one’s own beliefs and biases resulting from one’s individual experiences, training, and positionality as a researcher situated in an institution with a unique set of ideologies and biases—is critical. This includes being aware of assumptions about what the intervention could or should look like and what should help the end user, as well as being open to alternatives. Although reflexivity is important throughout all phases of the research project, it is essential during the formative work that ultimately shapes the data collected. The openness of UCD during this phase may allow for the identification of new, highly attuned treatment targets.

Once the research team has a good understanding of end-user needs, attention can be directed to ideating solutions. Ideating in this context means considering how individuals want their needs to be addressed. The transition from needs to ideating solutions can occur in the context of a single session through the pairing of UCD techniques (eg, interviews and scenarios), but it can also occur sequentially in a series of ≥2 sessions, depending on how much investigation is needed.

#### Example

The research team, which comprised clinical psychologists, psychiatrists, and clinical social workers, was interested in developing a service to support adolescents with suicidal thoughts or behaviors in their transition from inpatient to outpatient care. The first step was to elicit feedback from key stakeholders. Individual interviews were chosen to protect confidentiality, maximize comfort discussing SITB experiences, and alleviate concerns around youths’ susceptibility to effects of social desirability in group settings such as focus groups.

A total of 30 semistructured interviews were conducted with adolescents with lived experience with psychiatric hospitalization for suicidal ideation or behavior (10/30, 33%), their parents or guardians (10/30, 33%), and inpatient and outpatient care providers specializing in this population (10/30, 33%). The interviews included confirmatory and exploratory probes focused on stakeholders’ experiences with the transition between inpatient and outpatient care and impressions on how technology may support this transition for adolescents with SITB. The exploratory questions aimed to better understand the adolescents’ needs and preferences for timing, scope, and delivery of a smartphone app to support their care transition. The confirmatory questions probed the perceived importance of, and elicited contextual information on, core evidence-based treatment targets common to suicide prevention interventions [40-45].

Key findings across all stakeholder interviews included the need to focus on safety planning to smoothen the transitions in care. The perceived barriers to safety plan uptake during care transitions included lack of accessibility of paper-based safety plans, potential for adolescents to be too distressed to deploy the resources on their safety plan, and challenges with motivation to engage in treatment. The stakeholders viewed a smartphone app as an acceptable means to improve the accessibility of safety plans. Brief skills to reduce momentary experiences of distress that can act as a barrier to safety planning were considered essential, as were elements of motivational interviewing to bolster treatment adherence.

### Phase 2: Design

#### Overview

After the research team has a working understanding of the needs and preferences of end users and has worked with them to ideate possible solutions, the design feedback phase begins. The aim of this phase is to develop initial prototypes to share with end users to elicit information about acceptability and needs and further understand requirements of the intervention. This phase involves iterative prototype development and design activities with end users. Iteration is critical to allow for the gradual achievement of requirements through exploring options, pursuing new concepts, and refining prototypes. Studies show that research teams using design iteration outperform those with no iteration by producing better products that meet pre-established design requirements [46,47]. Qualitative feedback is analyzed and integrated into a progressively high-fidelity (or functional) prototype to be subjected to further feedback and evaluation. This phase usually concludes when the prototype seems to address the significant needs expressed by the participants.

#### Example

The research team aimed to create a smartphone app with safety planning and distress tolerance components that would be acceptable to adolescents with suicidal thoughts or behaviors based on the findings from the elicitation phase. They partnered with a private company with user interface expertise to draft a wish list for the desired app components. Next, the research team, user interface experts, and adolescent stakeholders with lived SITB experiences engaged in iterative development of app prototypes.

The research team decided to collect feedback in individual and group settings because the content was less sensitive (eg, focusing on the app services rather than SITB experiences) and a group format would better facilitate consensus on design aspects. Efforts were made to optimize the adolescents’ experiences in design sessions. To reduce burden, the design sessions were held in the building where the adolescents were treated after hospitalization. To increase engagement, the adolescents were recognized as experts on their own experiences and co-designers of the app.

The adolescent stakeholders met to provide feedback on app prototypes at 3 time points, with 5-8 adolescents participating at each time point. This process began with low-fidelity wireframes (paper-based prototypes) that provided an initial flow through the app and ended with a high-quality prototype (working digital product). Storyboarding, which involves a visual narrative to convey the function and purpose of the app, was used to obtain stakeholder feedback. The adolescents were asked to consider the acceptability of anticipated app components (eg, mood-monitoring content, activities, function, and flow) during moments of distress. Parallel prototyping, wherein the adolescents were provided with similar variants of prototypes that differed by look and feel as well as conceptual elements, was also used to solidify color choices, design schema, and the app name. The researchers summarized the feedback and incorporated it into the design of the improved prototype at the end of each of the 3 iterations. The prototype was considered final when, through consensus, the research team determined that the themes from qualitative feedback and the scores from quantitative usability questionnaires (eg, Usefulness, Satisfaction, and Ease of Use Scale [48]) indicated a sufficient level of satisfaction [48]. The app was assessed to be safe for evaluative testing by the research team because it met best practice standards for safety planning and incorporated brief evidence-based interventions for youths with SITB (eg, distress tolerance skills).

Key findings from this phase included the importance of considering every aspect of design (eg, cognitive burden, availability and number of coping resources, and inclusion of appropriate crisis resources) from the mindset of an adolescent actively experiencing distress. The adolescents had low tolerance for app functions that were confusing or ambiguous, and they acknowledged that this could make them prematurely stop app use. They reported that the look and feel of the app could influence their mood. Some preferred light colors, which were perceived as hopeful and uplifting; others preferred darker colors that felt more authentic to their mood. Personalization was seen as critical to engagement. As such, the final design included safety plan content (eg, internal and external coping resources, reasons to live, and crisis contacts) that was personalized and interactive as well as a combination of dark and light colors to meet users’ esthetic preferences. Coping activities on the app included a combination of existing web-based content identified by the adolescents and clinicians who treat adolescents with suicidal thoughts or behaviors and the option for adolescents to add their own photos, videos, and websites.

### Phase 3: Usability Testing

#### Overview

The purpose of usability testing is to understand whether the product is usable and functional, as well as to identify additional refinements needed before the intervention is moved to the clinical trial phase. This phase, similar to the design phase, should involve iteration so that participant feedback can be integrated into the intervention before additional evaluations. A variety of approaches can be used during this phase, including in-laboratory testing and longitudinal field testing. In addition, this phase allows for mixed methods analysis of qualitative data from participant and researcher interactions, as well as quantitative data from standard usability scales. At the conclusion of this phase, the research team should have a usable and highly engaging intervention that meets end users’ needs.

#### Example

App usability was examined through individual sessions with 10 hospitalized adolescents before discharge as well as longitudinal field testing with 5 adolescents through an open pilot trial to ensure that they were able to navigate the app independently and to detect any remaining bugs. Specifically, the adolescents were asked to think aloud as they performed tasks such as composing their safety plan or accessing distress tolerance techniques. At the end of the session, the adolescents completed a quantitative assessment of usability. Usability metrics were good, and the adolescents had not identified major design issues; therefore, the app was ready for longitudinal testing. For the open trial, the adolescents received an orientation to the app while they were hospitalized, and they downloaded and used the app upon hospital discharge or shortly thereafter. They then used the app naturalistically during their transition to outpatient care. Brief telephone check-ins were conducted with the adolescents at 4 and 12 weeks after discharge. These interviews provided an opportunity for the adolescents to describe their use of the app, including contexts when the app was, and was not, helpful. This field testing revealed the need for additional changes to the app to improve technical function and engagement. Modifications were made before the launch of the RCT.

### Next Steps: Moving From Usability to Formal Evaluation of Treatment Targets

#### Overview

Once researchers have evidence of the acceptability and usability of the intervention, the final DMHI is ready to be tested in a pilot or fully powered clinical trial examining key clinical targets. During this phase, UCD methods are not often used, but they can be useful for further optimization and refinement based on problems encountered as an intervention is scaled up. This can be done in several ways, most often through including usability measures as secondary outcomes in RCTs and conducting interviews to get feedback after treatment. Another method for incorporating lived experience to adapt and refine the intervention through the trial process is suggested in the Accelerated Creation-to-Sustainment model [49]. Hybrid trials that account for the ever-evolving nature of technology can offer a more flexible and iterative approach to trial procedures and focus on optimization, effectiveness, and implementation in real-world settings [49,50]. These trials have the potential to significantly address the research-to-practice gap by producing high-quality digital interventions in a timely manner.

#### Example

In addition to an analysis of primary and secondary treatment outcomes (eg, suicidal ideation and behavior), the RCT included an evaluation of app usability through exit interviews as well as usability and satisfaction questionnaires. The exit interviews probed about use of the app and its components and included questions to evaluate the extent to which the app addressed key barriers identified during the qualitative interviews, such as ability to reduce distress in the moment, use of core components of the safety plan, and motivation to engage with the app.

The findings from this RCT included overall app use rates of approximately 70% and good usability and satisfaction scores on the Computer System Usability Questionnaire [51] over the 6-month follow-up period after hospitalization. The exit interviews revealed that the end users felt that the app was effective in aiding their use of key safety plan components, including seeking social support, reaching out to crisis support hotlines, and reducing momentary distress through practicing skills. The exit interviews also revealed when adolescents opted not to use the app; their decision was often influenced by motivational factors that were sometimes external to their experience with using the app. For some, the app was viewed as part of the treatment they were receiving more broadly; when motivation to engage with treatment waned, so did interest in using the app. On the basis of this finding, some further adjustments were made in the research team’s subsequent trial (currently underway) to extend the use of motivational interviewing strategies within the app and through a coached onboarding process to augment adolescents’ motivation for engagement with the app.

## Discussion

### Considerations and Future Directions

The aforementioned case example demonstrates how lived experience perspectives can be engaged in elicitation, design, and initial usability testing of a new DMHI for SITB. In addition to explaining the UCD process and providing a working example of what this process can look like in designing a DMHI product, we conclude by outlining promising opportunities to integrate lived experiences in research processes that run parallel to intervention development and in emerging areas of SITB research focus. We discuss the use of UCD in (1) establishing procedures related to participant safety and comfort, (2) developing data privacy protocols for just-in-time adaptive interventions, (3) reaching and engaging individuals who are not treatment seeking, and (4) developing protocols for implementation and sustainability.

### Establishing Procedures Related to Participant Safety and Comfort

UCD enables researchers to develop products as well as procedures and policies that align with end-user needs, concerns, and desires from the ground up. A promising extension of UCD for an SITB intervention is the development of protocols that ensure participant safety and comfort. This includes considerations related to risk management: (1) risks related to discomfort and privacy in the research process and (2) designing for safety. Researchers must be mindful of the burden that research activities may place on participants and be proactive in efforts to reduce burden. This includes explicitly addressing, and probing for, potential concerns around privacy, stigma, and unintended or unwanted disclosure. As topics relevant to SITB lived experience can be sensitive, researchers must plan for activities to be held at a time and place that is comfortable for participants and plan for distress management. This may include accommodations such as having at least two researchers present to conduct focus groups, with a nearby private room available for breaks or one-on-one engagement. Furthermore, staff members must have appropriate training and experience with suicide risk assessment and management.

Risk management also extends to considerations of the final intervention design itself. Effective risk management can influence whether users feel comfortable engaging with a tool or service; yet, risks are often considered without input from end users. The Trans Lifeline is a good example of a technology-enabled resource that adapted its services based on feedback from target users. The Trans Lifeline elected not to engage in nonconsensual active rescues after feedback from a 2015 survey with 800 transgender individuals [52]. Fear of such rescues, harm from encounters with law enforcement, and involuntary psychiatric hospitalization were identified as key barriers to crisis hotline use. Although crisis services can provide highly effective coping skills and emotion regulation tools that reduce the risk of a suicide attempt [53], certain interventions such as deploying police to respond to mental health crises may be harmful and reduce engagement and efficacy. Understanding the needs of constituents and the responses that communities find most beneficial and least harmful enables the tailoring of services, protocols, and systems that effectively manage suicide risk in alignment with the goals, values, rights, and dignity of the end users.

### Developing Data Privacy Protocols for Just-in-Time Adaptive Interventions

There are unique privacy considerations when collecting data from vulnerable populations such as individuals with SITB. As interest in the field has turned to personalized, adaptive, and in-the-moment interventions for suicide prevention and NSSI reduction [54], this necessitates the use of sophisticated passive sensing and self-report methods (eg, EMA). Given the granularity of such data, it becomes ever more important to consider privacy protocols that are aligned with end-user needs and ethics.

Discussions on how to engage individuals more meaningfully in the process of collecting and using such granular data already exist in the privacy literature and can be useful in SITB intervention. For example, Shilton [55] describes a “participatory sensing” approach where “participants are not just subjects of data collection, but take the role of investigators (when they collect data to participate in self-analytic applications) or co-investigators (when they contribute their data to larger research initiatives).” This means that individuals with lived SITB experience would have input into how, and what types of, data are collected. Participants might express comfort with certain types of data being collected passively, without their knowledge, if it could inform in-the-moment interventions. There may also be contingencies around collection—perhaps passive data collection is acceptable if users or their clinicians (if an augmentative service) get meaningful feedback [56]. There may also be contingencies around collection—perhaps passive data collection is acceptable if users or their clinicians (if an augmentative service) receive useful feedback. End-user insights should also inform how the data are processed and used by researchers and clinicians.

Although just-in-time interventions hold significant promise for SITB, there are also ethical concerns around managing participant safety and participant burden [54]. This work also tends to focus on individuals with lived experience as research subjects (eg, producers of data), rather than as collaborative informants or experts. We see UCD techniques as a useful complement for mitigating some challenges associated with EMA and passive data approaches. For example, a chief concern with EMA is that it may increase participant distress and burden. By conducting formative work, including surveys among individuals with lived experience and feasibility studies with different EMA timetables, researchers can identify appropriate intervention schedules that are comfortable for participants and meet their needs. For an example of formative work exploring the feasibility of a particular method of elicitation (web-based focus groups) among young people with suicidal thoughts, see the study by Han et al [57]. In this work, the authors examine participants’ comfort with, and desire to use, a web-conferencing technology for future research on suicide prevention. Incorporating lived experience in the development, testing, and implementation of just-in-time interventions and related study procedures such as data privacy protocols can reduce the likelihood of disengagement resulting from the intervention being burdensome and ensure that the intervention fits into participants’ lives and that data management processes are acceptable to participants or users.

In sum, when designing interventions that collect increasingly nuanced and granular health and mental health data, it is critical to consider the risks and how individuals perceive these risks, as well as proactively consider and address ethical concerns that may arise [58].

### Reaching and Engaging Individuals Who Are Not Treatment Seeking

UCD methods may be particularly valuable in the development of DMHIs for individuals not currently engaged in, or unlikely to engage in, formal mental health services. These individuals are not only underserved in the sense that they are not receiving services, but also their needs and goals are not well understood. Existing interventions available to this population may be inaccessible; may have been ineffective, leading to a discontinuation of services; or may not be aligned with their unique goals. Low rates of SITB or NSSI disclosure, often on account of fears of stigma or hostile risk management [5,59-61], complicate efforts to design resources for this group. A promising method for engaging a subset of this population is through web-based forums and social media sites where discussions of SITB occur regularly. Web-based activity is high among this population, in part because of the relative anonymity it affords [62-64], and UCD methods are flexible enough to meet and engage individuals in spaces where they are most comfortable. For example, recruitment can take place entirely on the web, such as through web-based forums [65], and remote UCD techniques can be used for all phases of the UCD process. Elicitation interviews can be conducted through telephone or texting, design feedback activities can occur through asynchronous anonymous focus groups, and usability testing can similarly be conducted through synchronous feedback sessions.

In sum, the potential to develop tools that meet the needs of individuals not currently treatment engaged is promising because it affords the possibility for individuals who are not interested in, or comfortable with, disclosing SITB to professionals to get support and services, and UCD provides a set of techniques to do so.

### Developing Protocols for Implementation and Sustainability

The value of UCD can also be extended to the development of protocols for implementation and sustainability. For DMHIs that are meant to augment, or work in conjunction with, formal treatment and service settings, researchers must engage clinicians and staff early on because the DMHI must fit into their workflows. For stand-alone DMHIs, a detailed plan for disseminating and advertising the product as well as a plan for any needed maintenance through periodic usability tests are needed. Early engagement of key stakeholders can ensure that the plan or protocol will meet their needs and is feasible given their resources and constraints. For a review and example of how UCD can be leveraged to improve implementation, see the studies by Lyon et al [37] and Dopp et al [66].

### Conclusions

UCD foregrounds key stakeholders with lived experience in DMHI design and in doing so may increase acceptability and engagement of interventions and illuminate intervention targets that are not readily apparent in existing theoretical frameworks or risk models. Early elicitation can increase researchers’ understandings of needs, preferences, and circumstances surrounding when and how stakeholders want to interact with interventions, digital devices, and care systems. Design activities facilitate ideation on desired DMHI components and ensure that stakeholders’ expertise and experience are incorporated into the final DMHI. Usability testing ensures that the final DMHI is perceived to be usable, useful, and acceptable to the population it will ultimately serve. UCD consists of a well-developed set of methods that have been broadly applied to address problems in many fields. We argue that this set of methods can help researchers address specific challenges to SITB interventions by providing a systematic process for invoking the lived experience of end users in research on the design, development, and evaluation of new interventions.

## Abbreviations

DMHI: digital mental health intervention
EMA: ecological momentary assessment
NSSI: nonsuicidal self-injury
RCT: randomized controlled trial
SITB: self-injurious thoughts and behaviors
UCD: user-centered design

## References

[1] Curtin S, Warner M, Hedegaard H (2016). Increase in suicide in the United States, 1999-2014. NCHS Data Brief.

[2] Swannell SV, Martin GE, Page A, Hasking P, St John NJ (2014). Prevalence of nonsuicidal self-injury in nonclinical samples: systematic review, meta-analysis and meta-regression. Suicide Life Threat Behav.

[3] Mortier P, Cuijpers P, Kiekens G, Auerbach RP, Demyttenaere K, Green JG, Kessler RC, Nock MK, Bruffaerts R (2018). The prevalence of suicidal thoughts and behaviours among college students: a meta-analysis. Psychol Med.

[4] Mortier P, Auerbach RP, Alonso J, Bantjes J, Benjet C, Cuijpers P, Ebert DD, Green JG, Hasking P, Nock MK, O'Neill S, Pinder-Amaker S, Sampson NA, Vilagut G, Zaslavsky AM, Bruffaerts R, Kessler RC, WHO WMH-ICS Collaborators (2018). Suicidal thoughts and behaviors among first-year college students: results from the WMH-ICS project. J Am Acad Child Adolesc Psychiatry.

[5] Michelmore L, Hindley P (2012). Help-seeking for suicidal thoughts and self-harm in young people: a systematic review. Suicide Life Threat Behav.

[6] Whitlock J, Eckenrode J, Silverman D (2006). Self-injurious behaviors in a college population. Pediatrics.

[7] Ammerman BA, Wilcox KT, O'Loughlin CM, McCloskey MS (2021). Characterizing the choice to disclose nonsuicidal self-injury. J Clin Psychol.

[8] Hermes ED, Lyon AR, Schueller SM, Glass JE (2019). Measuring the implementation of behavioral intervention technologies: recharacterization of established outcomes. J Med Internet Res.

[9] Schueller S, Tomasino K, Mohr DC (2016). Integrating human support into behavioral intervention technologies: the efficiency model of support. Clin Psychol Sci Pract.

[10] Arshad U, Gauntlett J, Husain N, Chaudhry N, Taylor PJ, Farhat-Ul-Ain (2020). A systematic review of the evidence supporting mobile- and internet-based psychological interventions for self-harm. Suicide Life Threat Behav.

[11] Fox KR, Huang X, Guzmán EM, Funsch KM, Cha CB, Ribeiro JD, Franklin JC (2020). Interventions for suicide and self-injury: a meta-analysis of randomized controlled trials across nearly 50 years of research. Psychol Bull.

[12] Graham AK, Lattie EG, Mohr DC (2019). Experimental therapeutics for digital mental health. JAMA Psychiatry.

[13] Torous J, Lipschitz J, Ng M, Firth J (2020). Dropout rates in clinical trials of smartphone apps for depressive symptoms: a systematic review and meta-analysis. J Affect Disord.

[14] Torous J, Michalak EE, O'Brien HL (2020). Digital health and engagement-looking behind the measures and methods. JAMA Netw Open.

[15] Hom MA, Podlogar MC, Stanley IH, Joiner TE (2017). Ethical issues and practical challenges in suicide research. Crisis.

[16] Lewis SP, Hasking P (2019). Putting the "Self" in self-injury research: inclusion of people with lived experience in the research process. Psychiatr Serv.

[17] (2014). The Way Forward: Pathways to hope, recovery, and wellness with insights from lived experience. Suicide Attempt Survivors Task Force of the National Action Alliance for Suicide Prevention.

[18] Kennard BD, Goldstein T, Foxwell AA, McMakin DL, Wolfe K, Biernesser C, Moorehead A, Douaihy A, Zullo L, Wentroble E, Owen V, Zelazny J, Iyengar S, Porta G, Brent D (2018). As Safe as Possible (ASAP): a brief app-supported inpatient intervention to prevent postdischarge suicidal behavior in hospitalized, suicidal adolescents. Am J Psychiatry.

[19] Kennard BD, Biernesser C, Wolfe KL, Foxwell AA, Craddock Lee SJ, Rial KV, Patel S, Cheng C, Goldstein T, McMakin D, Blastos B, Douaihy A, Zelazny J, Brent DA (2015). Developing a brief suicide prevention intervention and mobile phone application: a qualitative report. J Technol Hum Serv.

[20] Rex Hartson H (1998). Human–computer interaction: interdisciplinary roots and trends. J Syst Softw.

[21] Lyon A, Coifman J, Cook H, McRee E, Liu FF, Ludwig K, Dorsey S, Koerner K, Munson SA, McCauley E (2021). The Cognitive Walkthrough for Implementation Strategies (CWIS): a pragmatic method for assessing implementation strategy usability. Implement Sci Commun.

[22] Mullaney T, Pettersson H, Nyholm T, Stolterman E (2012). Thinking beyond the cure: a case for human-centered design in cancer care. Int J Design.

[23] Lyon A, Comtois K, Kerns S, Landes S, Lewis C (2020). Closing the science–practice gap in implementation before it widens. Implementation Science 3.0.

[24] Lyon AR, Bruns EJ (2019). User-centered redesign of evidence-based psychosocial interventions to enhance implementation-hospitable soil or better seeds?. JAMA Psychiatry.

[25] Stiles-Shields C, Montague E, Lattie EG, Schueller SM, Kwasny MJ, Mohr DC (2017). Exploring user learnability and learning performance in an app for depression: usability study. JMIR Hum Factors.

[26] Weinheimer EA, Chang A, Neubert SW, Wildes JE, Graham AK (2020). Past, current, and future willingness to engage with treatment targets: applying user-centered design to inform the design of a mobile behavioral intervention. Int J Eat Disord.

[27] Graham AK, Greene CJ, Kwasny MJ, Kaiser SM, Lieponis P, Powell T, Mohr DC (2020). Coached mobile app platform for the treatment of depression and anxiety among primary care patients: a randomized clinical trial. JAMA Psychiatry.

[28] Čuš A, Edbrooke-Childs J, Ohmann S, Plener PL, Akkaya-Kalayci T (2021). "Smartphone Apps Are Cool, But Do They Help Me?": a qualitative interview study of adolescents' perspectives on using smartphone interventions to manage nonsuicidal self-injury. Int J Environ Res Public Health.

[29] Honary M, Bell B, Clinch S, Vega J, Kroll L, Sefi A, McNaney R (2020). Shaping the design of smartphone-based interventions for self-harm. Proceedings of the 2020 CHI Conference on Human Factors in Computing Systems.

[30] Dimeff LA, Jobes DA, Chalker SA, Piehl BM, Duvivier LL, Lok BC, Zalake MS, Chung J, Koerner K (2020). A novel engagement of suicidality in the emergency department: virtual collaborative assessment and management of suicidality. Gen Hosp Psychiatry.

[31] Bush NE, Dobscha SK, Crumpton R, Denneson LM, Hoffman JE, Crain A, Cromer R, Kinn JT (2015). A Virtual Hope Box smartphone app as an accessory to therapy: proof-of-concept in a clinical sample of veterans. Suicide Life Threat Behav.

[32] Larsen ME, Shand F, Morley K, Batterham PJ, Petrie K, Reda B, Berrouiguet S, Haber PS, Carter G, Christensen H (2017). A Mobile Text Message Intervention to Reduce Repeat Suicidal Episodes: Design and Development of Reconnecting After a Suicide Attempt (RAFT). JMIR Ment Health.

[33] Conducting research with participants at elevated risk for suicide: considerations for researchers. National Institutes of Mental Health.

[34] Pisani AR, Wyman PA, Mohr DC, Perrino T, Gallo C, Villamar J, Kendziora K, Howe GW, Sloboda Z, Brown CH (2016). Human subjects protection and technology in prevention science: selected opportunities and challenges. Prev Sci.

[35] Nock MK, Kleiman EM, Abraham M, Bentley KH, Brent DA, Buonopane RJ, Castro-Ramirez F, Cha CB, Dempsey W, Draper J, Glenn CR, Harkavy-Friedman J, Hollander MR, Huffman JC, Lee HI, Millner AJ, Mou D, Onnela J, Picard RW, Quay HM, Rankin O, Sewards S, Torous J, Wheelis J, Whiteside U, Siegel G, Ordóñez AE, Pearson JL (2021). Psychiatr Res Clin Pract.

[36] (2018). Suicide reporting recommendations. American Association of Suicidology.

[37] Lyon AR, Brewer SK, Areán PA (2020). Leveraging human-centered design to implement modern psychological science: return on an early investment. Am Psychol.

[38] Czyz EK, Arango A, Healy N, King CA, Walton M (2020). Augmenting safety planning with text messaging support for adolescents at elevated suicide risk: development and acceptability study. JMIR Ment Health.

[39] Czyz E, King C, Prouty D, Micol V, Walton M, Nahum-Shani I (2021). Adaptive intervention for prevention of adolescent suicidal behavior after hospitalization: a pilot sequential multiple assignment randomized trial. J Child Psychol Psychiatry.

[40] Rossouw TI, Fonagy P (2012). Mentalization-based treatment for self-harm in adolescents: a randomized controlled trial. J Am Acad Child Adolesc Psychiatry.

[41] Pineda J, Dadds MR (2013). Family intervention for adolescents with suicidal behavior: a randomized controlled trial and mediation analysis. J Am Acad Child Adolesc Psychiatry.

[42] Esposito-Smythers C, Spirito A, Kahler CW, Hunt J, Monti P (2011). Treatment of co-occurring substance abuse and suicidality among adolescents: a randomized trial. J Consult Clin Psychol.

[43] Brown GK, Ten Have T, Henriques GR, Xie SX, Hollander JE, Beck AT (2005). Cognitive therapy for the prevention of suicide attempts: a randomized controlled trial. JAMA.

[44] Stanley B, Brown G, Brent DA, Wells K, Poling K, Curry J, Kennard BD, Wagner A, Cwik MF, Klomek AB, Goldstein T, Vitiello B, Barnett S, Daniel S, Hughes J (2009). Cognitive-behavioral therapy for suicide prevention (CBT-SP): treatment model, feasibility, and acceptability. J Am Acad Child Adolesc Psychiatry.

[45] Rotheram-Borus MJ, Bradley J (1991). Triage model for suicidal runaways. Am J Orthopsychiatry.

[46] Dow S, Heddleston K, Klemmer S (2009). The efficacy of prototyping under time constraints. Proceedings of the seventh ACM conference on Creativity and cognition.

[47] Camburn B, Viswanathan V, Linsey J, Anderson D, Jensen D, Crawford R, Otto K, Wood K (2017). Design prototyping methods: state of the art in strategies, techniques, and guidelines. Des Sci.

[48] Lund A (2001). Measuring usability with the USE questionnaire. Usab Interface.

[49] Mohr DC, Lyon AR, Lattie EG, Reddy M, Schueller SM (2017). Accelerating digital mental health research from early design and creation to successful implementation and sustainment. J Med Internet Res.

[50] Mohr DC, Schueller SM, Riley WT, Brown CH, Cuijpers P, Duan N, Kwasny MJ, Stiles-Shields C, Cheung K (2015). Trials of intervention principles: evaluation methods for evolving behavioral intervention technologies. J Med Internet Res.

[51] Lewis JR (1995). IBM computer usability satisfaction questionnaires: psychometric evaluation and instructions for use. Int J Human Comput Interac.

[52] Why no non-consensual active rescue?. Trans Lifeline.

[53] Gould M, Lake A, Galfalvy H, Kleinman M, Munfakh J, Wright J, McKeon R (2018). Follow-up with callers to the national suicide prevention lifeline: evaluation of callers' perceptions of care. Suicide Life Threat Behav.

[54] Coppersmith D, Dempsey W, Kleinman E, Bentley K, Murphy S, Nock M Just-in-time adaptive interventions for suicide prevention: promise, challenges, and future directions. PsyArXiv Preprint.

[55] Shilton K (2009). Four billion little brothers?: privacy, mobile phones, and ubiquitous data collection. Commun ACM.

[56] Nicholas J, Shilton K, Schueller SM, Gray EL, Kwasny MJ, Mohr DC (2019). The role of data type and recipient in individuals' perspectives on sharing passively collected smartphone data for mental health: cross-sectional questionnaire study. JMIR Mhealth Uhealth.

[57] Han J, Torok M, Gale N, Wong QJ, Werner-Seidler A, Hetrick SE, Christensen H (2019). Use of web conferencing technology for conducting online focus groups among young people with lived experience of suicidal thoughts: mixed methods research. JMIR Ment Health.

[58] Mulvenna MD, Bond R, Delaney J, Dawoodbhoy FM, Boger J, Potts C, Turkington R (2021). Ethical issues in democratizing digital phenotypes and machine learning in the next generation of digital health technologies. Philos Technol.

[59] Whitlock J, Muehlenkamp J, Purington A, Eckenrode J, Barreira P, Baral Abrams G, Marchell T, Kress V, Girard K, Chin C, Knox K (2011). Nonsuicidal self-injury in a college population: general trends and sex differences. J Am Coll Health.

[60] Fortune S, Sinclair J, Hawton K (2008). Help-seeking before and after episodes of self-harm: a descriptive study in school pupils in England. BMC Public Health.

[61] Whitlock JL, Powers JL, Eckenrode J (2006). The virtual cutting edge: the internet and adolescent self-injury. Dev Psychol.

[62] Lewis SP, Rosenrot SA, Messner MA (2012). Seeking validation in unlikely places: the nature of online questions about non-suicidal self-injury. Arch Suicide Res.

[63] Rodham K, Gavin J, Lewis SP, St Dennis JM, Bandalli P (2013). An investigation of the motivations driving the online representation of self-injury: a thematic analysis. Arch Suicide Res.

[64] Lewis SP, Michal NJ (2016). Start, stop, and continue: preliminary insight into the appeal of self-injury e-communities. J Health Psychol.

[65] Smith DM, Lipson SM, Wang SB, Fox KR (2021). Online methods in adolescent self-injury research: challenges and recommendations. J Clin Child Adolesc Psychol.

[66] Dopp AR, Parisi KE, Munson SA, Lyon AR (2020). Aligning implementation and user-centered design strategies to enhance the impact of health services: results from a concept mapping study. Implement Sci Commun.

